# Clot Retraction: Cellular Mechanisms and Inhibitors, Measuring Methods, and Clinical Implications

**DOI:** 10.3390/biomedicines9081064

**Published:** 2021-08-21

**Authors:** Ellen E. Jansen, Matthias Hartmann

**Affiliations:** 1 Clinic for Operative Dentistry, Periodontology and Preventive Dentistry, RWTH Aachen University, 52074 Aachen, Germany; ejansen@ukaachen.de; 2 Klinik für Anästhesiologie und Intensivmedizin, Universitätsklinikum Essen, Universität Duisburg-Essen, 45122 Essen, Germany

**Keywords:** clot retraction, platelet force development, αIIbβ3 receptor

## Abstract

Platelets have important functions in hemostasis. Best investigated is the aggregation of platelets for primary hemostasis and their role as the surface for coagulation leading to fibrin- and clot-formation. Importantly, the function of platelets does not end with clot formation. Instead, platelets are responsible for clot retraction through the concerted action of the activated αIIbβ3 receptors on the surface of filopodia and the platelet’s contractile apparatus binding and pulling at the fibrin strands. Meanwhile, the signal transduction events leading to clot retraction have been investigated thoroughly, and several targets to inhibit clot retraction have been demonstrated. Clot retraction is a physiologically important mechanism allowing: (1) the close contact of platelets in primary hemostasis, easing platelet aggregation and intercellular communication, (2) the reduction of wound size, (3) the compaction of red blood cells to a polyhedrocyte infection-barrier, and (4) reperfusion in case of thrombosis. Several methods have been developed to measure clot retraction that have been based on either the measurement of clot volume or platelet forces. Concerning the importance of clot retraction in inborn diseases, the failure of clot retraction in Glanzmann thrombasthenia is characterized by a bleeding phenotype. Concerning acquired diseases, altered clot retraction has been demonstrated in patients with coronary heart disease, stroke, bronchial asthma, uremia, lupus erythematodes, and other diseases. However, more studies on the diagnostic and prognostic value of clot retraction with methods that have to be standardized are necessary.

## 1. Introduction

Coagulation is characterized by the formation of a fibrin mesh which includes platelets as well as red blood cells and leucocytes. The aim of clot formation is the achievement of hemostasis and tissue repair [1]. Platelets have an active role in this process, releasing hundreds of mediators and influencing clot strength and volume. Indeed, the platelet αIIbβ3 receptors, which control a contractile apparatus via complex signal transduction pathways, bind and pull at the fibrin strands to reduce the volume of the clot. Clot retraction starts immediately with fibrin formation and the activation of platelets and lasts for several hours. During primary hemostasis, clot retraction brings the platelets in close contact, hindering the diffusion of signaling molecules and thus favoring their accumulation to allow cell–cell communication that is similar to a synapse [2,3]. Moreover, the volume reduction of the clot allows for reperfusion in the case of thrombosis, the tight packing of the red blood cells (named polyhedrocytes) hinders the entrance of pathogens, and the retraction of the wound edges facilitates healing [4,5].

In the present review on clot retraction, we review cell physiology, known pharmacological targets, the various but not standardized methods of measurement, and the sparse findings in human diseases.

## 2. Cell Biology of Clot Retraction

### 2.1. The αIIbβ3 Receptor

The integrin αIIbβ3 is a heterodimer expressed at a high level on the surface of the platelets. Approximately 50,000–100,000 copies can be found on a single platelet [6,7]. αIIbβ3 binds to fibrinogen, fibrin, and the von Willebrand factor and plays an important role in clot retraction as well as platelet aggregation, hemostasis, and even cancer progression. The integrin is capable of bidirectional signaling. Platelet activation, e.g., with thrombin, collagen, or thromboxane A2 induces a conformational change of the αIIbβ3 receptor from the low-affinity to the high-affinity state of the extracellular domain. In turn, agonist-binding to the αIIbβ3 receptor activates outside-in signaling, leading to clot retraction as well as stable adhesion, spreading, and irreversible aggregation [3,6].

Besides clot retraction, the binding of fibrinogen to activated αIIbβ3 receptors is of enormous importance for primary hemostasis. Interactions are facilitated by the release of the intracellular tether of the αIIbβ3 receptors, allowing the exposure of multiple bindings sites for fibrinogen as well as von Willebrand factor, possibly via release from cytoskeletal actin components [8].

### 2.2. Signal Transduction of αIIbβ3

Inside-out signaling is achieved by the activation of G protein-coupled receptors via the agonists thrombin, thromboxane A2, ADP, epinephrine, and collagen and subsequent phospholipase C, β, or γ (dependent on the agonist) activation. Phospholipase C, in turn, hydrolyzes phosphatidylinositol (4,5)-bisphosphate into diacylglycerol and inositol (1,4,5) triphosphate. As a consequence, the guanine nucleotide exchange factor activates the GTPase RAS-related protein 1. RAP1 binds to RAP1–GTP-interacting adaptor molecule, talin, and kindlin, and the resulting complex activates αIIbβ3.

**Figure 1:**
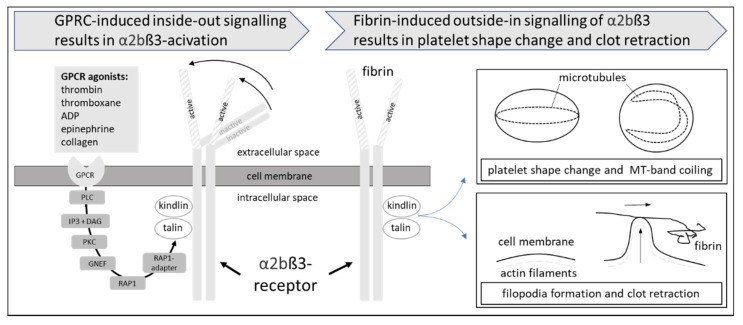
Inside-out and outside-in signaling of the platelet αIIbβ3-receptor. Inside-out signaling is defined as the activation of the extracellular part of the αIIbβ3-receptor induced by intracellular signal transduction events including G-protein coupled receptors (GPCR), phospholipase C (PLC) and the second messengers diacylglcerol (DAG) and inosittrisphosphate (IP3), protein kinase C (PKC), RAS-related protein 1 (RAP1), RAP1-adapter, talin, and kindlin. Outside-in signaling consists of fibrin-binding to the activated αIIbβ3 receptor, subsequent activation of complex intracellular signal transduction pathways (not shown), leading to a shape change induced by the microtubule system as well as filopodia formation and clot retraction induced by actin reorganization.

Outside-in signaling of the αIIbβ3 receptor occurs upon the binding of fibrin or fibrinogen to the receptor. While many of the components used for outside-in signaling are different from those used for inside-out signaling, talin and kindlin are used for both signaling directions. The activation of αIIbβ3 promotes the activation of the non-receptor kinase Src, which dissociates from the cytoplasmic part of the receptor after cleavage by the thiol protease calpain and, in turn, activates multiple enzymes and signaling proteins. Talin and kindlin are involved in the coupling of actin to the αIIbβ3 receptors. Details of the complex signal transduction cascades responsible for the bidirectional signal transduction of the αIIbβ3 receptor, which are beyond the scope of the present review, have recently been reviewed in detail [6]. The inside-out and outside-in signaling of αIIbβ3 are visualized in Figure 1.

### 2.3. The Contractile Apparatus of Platelets

Subsequent to the activation of platelets, the contractile apparatus of the platelet changes the shape of the cell from a discoid to a spheroid shape and is responsible for the so-called spreading, the formation of filopodia and lamellipodia as well as clot retraction and migration [9]. Important constituents of the contractile apparatus are the microtubules and actin filaments. The microtubules build an intracellular ring called the microtubule band, which is located in the equator region of the platelets [10]. The configuration is responsible for the disc shape of the resting platelets. Upon activation of the platelet, the microtubule band conformation changes driven by the molecular motor dynein. The so-called coiling of the microtubule band results in a platelet taking on a spheroid form. Simultaneously, platelet activation induces a marked reorganization of actin, leading to the formation of lamellipodia and filopodia. Reorganized actin together with the molecular motor myosin IIa and the microtubule band as the structural support are capable of generating and transducing the force for clot retraction and migration to the extracellular space [11,12].

## 3. Pharmacologic Inhibition of Clot Retraction

To affect clot retraction in an experimental setting, the signaling of the αIIbβ3 receptor or the platelet contractile apparatus can be targeted. Accordingly, clot retraction has been demonstrated to be inhibited at several targets of the signal-transduction pathway.

### 3.1. Pharmakons Affecting Clot Retraction in Humans

Tirofiban, abciximab, and eptifibatide are partial αIIbβ3 agonists used in humans to prevent death and ischemic complications in coronary heart disease. The drugs markedly inhibit clot retraction but may activate αIIbβ3 and the binding of physiological substrates due to their partial agonistic action [13,14,15]. However, the pure peptide antagonist Hr10 and a tirofiban analog in an experimental setting did not inhibit clot retraction [16]. Moreover, clot retraction is inhibited in the presence of the two phosphodiesterase inhibitors theophylline and papaverine. The alkaloids are clinically used in bronchial asthma and arterial graft spasms in cardiac surgery, respectively [17]. The mechanism of action has not been clarified, but cross talk between GPCR signaling and αIIbβ3 can be suggested. In both probands and patients with chronic myeloid leukemia, treatment with dasatinib, a multikinase inhibitor, inhibits convulsin-induced phosphatidylserine exposure, αIIbβ3-activation, thrombin formation, clot retraction, and thrombus volume [18]. As the mechanism, the inhibition of collagen signaling via the inhibition of Src, known to be involved in outside-in signaling, caused by an off-target effect of dasatinib, has been demonstrated.

Carr et al. demonstrated, that thrombin is necessary for clot retraction using batro-xobin, which induces fibrin formation without thrombin generation [19]. Accordingly, several clinically used agents including the thrombin binding proteins thrombomodulin and antithrombin as well as the thrombin antagonists heparin, hirudin, lepirudin, bivalirudin, and argatroban inhibited clot retraction [19,20]. Moreover, the experimental agents P-PACK II (d-Phenylalanyl-L-Phenylalanylarginine- chloro-methyl ketone 2 HCl) and Thromstop (BNas-Gly-(pAM)Phe-Pip) inhibited clot retraction [19].

### 3.2. Drugs Affecting Clot Retraction in the Experimental Setting

At the level of the αIIbβ3 receptor, the crosslinking of platelet-associated fibrin fibers by the transglutaminase factor XIIIa is a prerequisite for outside-in signaling. Accordingly, clot retraction was abolished in factor XIII deficient mice, and it was inhibited by the hamster monoclonal antibody 7E9, which binds to the gamma chain of mouse fibrinogen [21,22]. Moreover, the inhibition of factor XIII with iodoacetamide, which alkylates factor XIIIa, and cystamine, a competitive inhibitor, inhibited clot retraction [23]. Both agents can only be used in the in vitro setting. Several agents binding at αIIbβ3 have been demonstrated to have differential effects on platelet aggregation and clot retraction. The RGDS peptide (Arg-Gly-Asp-Ser) and γ-400–411 dodecapeptide inhibit platelet aggregation without an effect on clot retraction [14,23]. Moreover, the monoclonal antibodies 10E5 and AP2 blocked aggregation but not clot retraction [13,24,25].

An early study investigating the platelet force development in an organ bath, demonstrated that clots relaxed in the presence of the mycotoxin cytochalasin D [26]. As the mechanism of action, the inhibitory effect of cytochalasin D on the actin polymerization of the microtubule band has been suggested [13].

Furthermore, ions can affect clot retraction: the reduction of extracellular Ca^2+^ reduced clot tension in an early study [26].

A partial inhibition of clot retraction was achieved by the inhibition of the Src-kinase involved in outside-in signaling with PP2 and PD173952 [27]. Inhibition of phosphotyrosine phosphatase using phenyl-arsine oxide and peroxovanadate markedly reduced clot retraction [13]. The involvement of phosphotyrosine phosphatases in the cytoskeletal reorganization necessary for clot retraction has been claimed to be the involved mechanism [28]. Interestingly, okadaic acid, a serin/threonine phosphatase inhibitor, also inhibited clot retraction, suggesting a complex regulation [28].

The contractile apparatus of platelets can be directly targeted by blebbistatin, which is a small molecule inhibitor of myosin IIa ATPase activity [21,23,27].

Perioxynitrite, an agent released during inflammation, has been demonstrated to inhibit clot retraction. An effect on mitochondrial energy production has been suggested as the mechanism [29].

Under physiological conditions, the activation of platelets leads to the activation of coagulation and fibrin formation as well as membrane scrambling via the activation of scramblases. Thus, platelets are activated and clot retraction is initiated in parallel. For some experimental settings, it might be interesting to induce coagulation without platelet activation and clot retraction. This aim can be achieved by reptilase (batroxobin), which was used to investigate the effect of thrombin and thrombin antagonists on clot retraction [19,30]. Thus, it was possible to determine the kinetics of clot retraction induced by thrombin in clots already formed by reptilase [31]. Membrane scrambling does not affect clot retraction, as evidenced by scramblase-deficient (TMEM16F deficient) mice, excluding the crosstalk of signaling pathways between both cellular events [32].

## 4. Measurement of Clot Retraction

**Figure 2:**
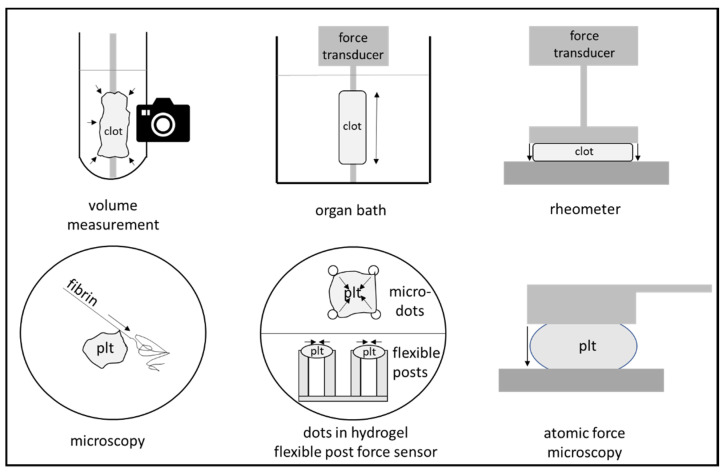
Methods for the measurement of clot retraction. Macro-methods (**upper row**) include the observation of the clot retracting in a vial, the measurement of force development of a molded clot in an organ bath, and the use of a rheometer. Micro-methods (see **lower row**) include the microscopic observation of platelet (plt) movement, the observation of traction of microdots in a hydrogel, or the flexion of flexible post force sensors as well as the use of atomic force microscopy.

Clot retraction is a phenomenon that can be seen in every clinical chemistry laboratory. For certain laboratory evaluations, blood samples are drawn in vials with thrombogenic surfaces. Coagulation and clot retraction lead to the separation of clot and the serum fraction, the latter of which is used for many analyses in clinical chemistry. In order to quantify clot retraction, several devices with different detection methods have been developed. Unfortunately, no device that is specifically developed for the measurement of clot retraction is currently available. For the determination of clot retraction, platelet rich plasma is most often used, but whole blood and isolated platelets reconstituted in an artificial medium can be used, too. Clot retraction can be quantified by the measurement of the decreasing clot volume or by the development of force by the platelets. The different methods are shown in Figure 2. Since clot retraction starts with the activation of platelets and the generation of fibrin and is long-lasting, clot reduction effects are often measured within 10 min to several hours.

### 4.1. Clot Volume (or Clot Area) as a Measure for Clot Retraction

The simplest method for the measurement of clot volume is the volumetric analysis. Coagulation of platelet rich plasma, blood, or isolated platelets (reconstituted in fibrinogen) is initiated by the addition of an activator (e.g., thrombin) in a vial. A wooden stick is placed in the vial. For the clot retraction measurement, the clot is removed after a certain time, and the serum volume is determined either gravimetrically or volumetrically [33]. As an alternative, the vial can be photographed at various time points to determine the time dependent changes to the clot area [33].

Moreover, a commercially available device, the Thrombodynamics Analyzer System (HemaCore, Moscow, Russia), has be used for an automated and continuous determination of the clot area [34]. The device uses an LED-light source, a cuvette, and measures the light transmission, taking images every 15 s via a CCD camera and the computational processing of the photographs. The intended use of the device is the measurement of clot formation and lysis as well as the measurement of thrombin generation in basic sciences. However, the device has been used for the measurement of clot retraction. Adhesion of the retracting clot to the cuvette wall was hindered by triton X100 during pretreatment, and the kinetic of retraction was evaluated after recalcification and the addition of thrombin from citrated whole blood samples.

All methods relying on clot volume are critically dependent on the blood preparation method that is used. When using whole blood, retraction is stopped when the red blood cells are compacted to polyhedral structures. Thus, the degree of clot retraction that can be measured is critically dependent on hematocrit and will normally be limited to about 50–60%. When platelet rich plasma is used, the retraction will proceed to about 80 to 90%. Moreover, the concentration of fibrin can be expected to influence clot retraction. Reconstitution experiments have demonstrated that an increase of fibrin from 1 mg/mL to 5 mg/mL was accompanied by a 60% reduction of clot retraction [23]. Results from clot retraction experiments using a volumetric method might not be comparable to clot area measurements, as the clot volume describes the three-dimensions of the clot and will therefore report bigger changes than the two-dimensional measurement of the surface area.

### 4.2. Microscopy for the Measurement of Clot Retraction

Advanced microscopic techniques allow the staining, direct visualization, and measurement of platelet action and give a detailed view on the action of the filopodia on the single cell level [35]. The shrinking of micro blood clots has been investigated in flow chambers [3].

A recently developed platelet contraction cytometer allows the measurement of the force development of individual platelets with a high-throughput technique [36]. For this method, 20,000 fluorescently conjugated fibrinogen dot pairs are embedded in several polyacrylamide hydrogels with different stiffnesses and are assembled in a microfluidic chamber. The force development of the platelets can be calculated from the movement of the dot pairs, which can be observed using super-microscopy techniques. This method demonstrates huge differences in force development and suggests different platelet populations in one patient.

Similarly, microscopic flexible post force sensor arrays have been used for the measurement of individual platelet forces [37]. Fibrinogen is absorbed to the tips of vertical posts made from polydimethylsiloxane. Platelets then settle to the tip, binding to the fibrinogen. The force development of the platelets can be calculated by the movements of the tips of the posts, which can be observed using microscopy. Repetitive measurements allow the determination of time dependency.

### 4.3. Force Development as a Measure for Clot Retraction

Measurement of the force caused by clot retraction is an alternative to the measurement of clot volume. In principle, blood or platelet rich plasma coagulating and retracting between two plates leads to the attraction of both plates (see Figure 2) [38]. Unfortunately, there is no specialized device available, but force development can be measured through the use of a commercially available rheometer.

Alternatively, a clot strip can be formed in a cylindrical tube and can be connected to a force transducer in an “organ bath”. Interestingly, drug diffusion is possible, and the (reversible) effects of drugs on clot retraction have been demonstrated with this method [26,39,40]. Typical isometric forces described in the literature are 1–2 mg per cm^2^, and the force development of a single platelet has been estimated to be about 20 nN [41].

Meanwhile, a miniaturized hemoretractometer has been developed to work with a 12.75 µL volume [42]. However, the device is not sold at present time. Besides developments such as micropost arrays and the high-throughput platelet-force cytometer (discussed under Section 4.2.), atomic force microscopy has been used for platelet force measurements at the single platelet level [36,37,43,44]. The contracting force of a single platelet was determined to be 29 nN with the latter method [44].

### 4.4. Point of Care Coagulation Devices for the Measurement of Clot Retraction

Rotational thrombelastometry (and thrombelastography) are devices that are often used in the operative setting and time dependently determine the firmness of a clot in whole blood samples. Technically, the hindrance of the back-and-forth movement of a rotating pin during clot formation is measured. The time course of clot firmness is the result of fibrin formation and the action of the platelets. Coagulation activation can be achieved by tissue factor or ellagic acid to activate the extrinsic or intrinsic coagulation systems, respectively (EXTEM test, INTEM test) [45]. Moreover, the effect of platelets on clot firmness can be inhibited by cytochalasin D, and the fibrinolytic system can be inhibited by tranexamic acid (FIBTEM test, APTEM test). A decrease in clot firmness with time (usual 60 min), the so called clot lysis index, has been suggested to be caused by clot retraction in the absence of fibrinolysis [46]. However, rotational thrombelastography has been demonstrated to be less sensitive than clot retraction measurements when platelet clot retraction was inhibited with abciximab [15]. In the thrombelastometry/thrombelastography literature, the missing decrease in clot firmness is associated with increased mortality in patients with sepsis, trauma, and liver transplantation. This finding has been explained as a fibrinolytic shutdown [47,48]. In a recent study on the association of the clot lysis index with mortality in liver transplantation patients, fibrinolysis was excluded as the reason for the observed association by means of the addition of tranexamic acid [49]. Differences in clot retraction might therefore explain the observed differences in the clot lysis index.

The sonoclot device uses a transducer vibrating at ultrasound frequency and measures the impedance during the clotting process. The impedance curve records an increase in the initial phase followed by a downward slope due to clot retraction. The sonoclot device has not been used to investigate clot retraction in a clinical setting.

### 4.5. Further Developments in Clot Retraction Assays

As outlined above, there are several different methods to measure clot retraction. It can be expected that there are important differences in the results obtained using these assays. Results may be affected by platelet count, red blood cell count, fibrinogen concentration, eventual defects in the coagulation system and inhibitors, the activator used as well as temperature and pH. Moreover, the rate of clot volume reduction as a measure for the speed of filopodia movement might not necessarily be closely correlated with the force development of platelets. Thus, further method comparison and improvements in assay standardization are necessary for clinical usage.

## 5. Clot Retraction in Human Diseases

### 5.1. Inborn Diseases Affecting Clot Retraction

Glanzmann thrombasthenia, described in 1918, leads to abolished clot retraction due to an inborn defect of the αIIbβ3 receptor [50]. Moreover, patients with Wiskott–Aldrich disease and May–Hegglin disorder (myosin IIa mutation) show a marked reductions of platelet forces, as measured with high-throughput platelet-contraction cytometry [36].

### 5.2. Acquired Diseases with Altered Clot Retraction

In patients with acute uremia due to kidney failure, clot retraction is greatly reduced to about 10% of the healthy probands [31]. Clot retraction increased in a patient with uremia after the infusion of DDAVP, most probably via the release of the von Willebrand factor from the endothelium [31].

Marked increases in platelet contractile force have been demonstrated in patients with coronary heart disease presenting for coronary artery bypass grafting in comparison to healthy probands. A two-fold increase was measured in patients with aspirin medication in comparison to healthy probands, and a four-fold increase was measured in those patients with contraindications for platelet inhibitors [51]. These results were confirmed in patients with coronary heart disease and chest pain [52]. During cardiac surgery, clot force development decreased with the duration of cardiopulmonary bypass, possibly induced by a reduction of the αIIbβ3 receptors on the platelet surface [53]. It is, however, important to emphasize, that there are multiple mechanisms leading to alterations of the coagulation system during cardiopulmonary bypass [54].

In ischemic stroke, a reduced clot retraction was noted when using an optical tracking system (thrombodynamics analyzer system, Moscow, Russia) in whole blood samples [34]. It cannot be concluded from the experiments that the contractile force of an individual platelet changed, as stroke is associated with a decrease in platelet count and an increase in fibrinogen (which is known to decrease clot retraction). However, whole blood retraction characteristics might affect clinical outcome in this setting [34].

Asthma has been shown to be associated with reduced clot retraction by approximately 50%, as demonstrated in a whole blood assay using repetitive photographs and evaluation via an imaging program [55]. In systemic lupus erythematodes, another inflammatory disease, clot retraction is decreased [56]. When the causal anti-dsDNA antibodies were applied to platelets from healthy probands, a clot retraction inhibition was observed, suggesting an autoantibody-mediated mechanism.

Clot retraction can also be affected by disturbances in the coagulation system, which lead to reduced thrombin formation and thus reduced platelet activation. Accordingly, many coagulation factor deficiencies including factor VII, VIII, XI, XIII as well as fibrinogen lead to a decrease in clot retraction [31]. Clot retraction in patients with bleeding without obvious reason (as based on conventional laboratory findings) was significantly reduced, as evidenced by high-throughput cytometry [36].

Many hematological diseases are associated with alterations in clot retraction. In patients with polycythemia vera, clot retraction, as determined by the determination of clot volume, is markedly inhibited, and the maximum amplitude of thrombelastometry does not reflect these changes [14]. In patients with sickle cell anemia, clot retraction, which was determined in whole blood samples with a volumetric assay, was reduced [23]. Moreover, clot retraction increased in anemia, as determined by the same volumetric method in that study. Interestingly, platelet force development increased with red blood cell count in the same study. The extent of clot retraction has recently been shown to be reduced by 53% in patients with sickle cell anemia with increased erythrocyte stiffness [57].

In case of organ ischemia, the flow arrest triggers clot retraction through the accumulation of ADP and thromboxane in the clot [58]. Furthermore, the ischemia-induced acidosis reduces further thrombosis by the inhibition of platelet functions and inhibits clot retraction in the further course [59].

## 6. Conclusions

Clot retraction is involved in many important physiological processes including hemostasis, wound healing, defense against microbes, and the resolution of thrombosis. Disturbances have been described in many common diseases (e.g., coronary heart disease and stroke). While there is ample knowledge on the signal transduction pathways and the contractile apparatus available, translational research is only beginning. Measurement standardization, commercially available devices, and clinical studies on the eventual diagnostic value are necessary. Finally, the possibility of pharmacological targeting should be evaluated in diseases with disturbed clot retraction.

## Data Availability

Does not apply for the present review.
